# Burnout among postgraduate doctors in Colombo: prevalence, associated factors and association with self-reported patient care

**DOI:** 10.1186/s12909-019-1810-9

**Published:** 2019-10-16

**Authors:** Beminihennedige Minuri S. Fernando, Dulani Lakmali Samaranayake

**Affiliations:** 1 Postgraduate Institute of Medicine, University of Colombo, Colombo, Sri Lanka; 2 Department of Community Medicine, Faculty of Medicine, University of Colombo, Colombo, Sri Lanka

**Keywords:** Burnout, Postgraduate doctors, Self-reported patient care

## Abstract

**Background:**

Postgraduate doctors are prone to burnout due to occupational and educational stressors. Sri Lankan situation is unknown. This study determines burnout among postgraduate doctors in Colombo: Prevalence, associated factors, and association with self-reported patient care.

**Methods:**

A cross-sectional study was conducted among 278 postgraduate doctors from eight specialties working in Colombo district, attached to the main postgraduate training institute for medical professionals. A self-administered questionnaire was used. It comprised of the Copenhagen Burnout Inventory and an author-developed questionnaire, which was used to assess, associated factors and self-reported patient care. Prevalence of burnout was calculated. Associations were analysed using chi-square and binary logistic regression.

**Results:**

The response rate was 88.1% (*n* = 245). The prevalence of personal, work-related and client-related burnout was 41.6% (95% CI = 35.5–47.8%), 30.6% (95% CI = 24.8–36.4%), 8.9% (95% CI = 5.4–12.5%) respectively.

Personal burnout was positively associated with, the trainee being a female, having a chronic disease, being involved in frequent unhealthy habits, having doctor parents, having home–work demands and having emotional demands. It was negatively associated with, having frequent healthy habits, being satisfied with skill development opportunities, and frequent use of deep studying. Work-related burnout was positively associated with, female gender, being involved in frequent unhealthy habits, having home–work demands and having emotional demands. It was negatively associated with, frequent use of deep methods of studying. Client-related burnout was positively associated with having emotional demands and negatively associated with being satisfied with training.

The frequent self-reported, suboptimal patient-care practices: poor communication, poor clinical practice, poor response to patient’s needs and poor communication during handing over were associated positively with client-related burnout.

**Conclusions:**

Most postgraduate doctors in Colombo have high personal and work-related burnout but client-related burnout is less. The factors associated with burnout need to be addressed by the programme managers of the postgraduate courses. Preventive measures should be introduced to reduce burnout among future postgraduate trainees of Colombo.

## Background

Burnout is defined as ‘a psychological condition of emotional exhaustion, depersonalisation and reduced personal accomplishment occurring in people persistently exposed to emotional and interpersonal stressors at work’ [1]. Postgraduate trainees in medicine are prone to burnout due to stressors of work and training [2]. It can have many undesirable effects on the training, education and patient care of the trainees, as well as on the overall health system [3].

The global prevalence of burnout among postgraduate doctors is shown vary from 27 to 75% [4–6] . It is associated with multiple stressors in the personal and family life [4, 7–11], work life [6, 8, 12–16], as well as factors related to their education, training and the learning environment [10, 15]. Burnout compromises postgraduate doctors’ quality of life, productivity, performance, ability to acquire knowledge and their satisfaction regarding the training programme [17, 18].

Postgraduate trainees’ or residents’ burnout has been widely researched worldwide. A recent meta-analysis of 47 studies shows that the evidence is concentrated in the Western countries and is limited from the South Asian settings [5]. A literature review by Bangal further highlights the scarcity of studies from India [19]. A recent editorial of the Lancet highlights the lack of evidence on burnout from Asian and lower middle-income countries [20]. Recent studies from the South Asian region are restricted by the small sample sizes and by being limited to a single institution or discipline [21–25]. No studies were found on postgraduate doctors’ burnout from the Sri Lankan context. In South Asian countries, a large proportion of the population is dependent on the public-funded health care services, which are characterized by a high patient load per doctor. Postgraduate doctors in these settings are therefore exposed to a high workload and occupational stress.

Sri Lanka has a strong public-funded health care system, serving majority of the population. Doctors employed in the public sector are provided postgraduate training by a government-funded institute. This training process produces specialists for the requirements of the government health system. Therefore, achieving the optimum training outcomes in these postgraduate trainees is important from the point-of view of the country as well as that of the trainees. It is a concern whether these young professionals are prone to burnout, due to excessive educational and patient care demands, and this may hinder them from achieving the optimum outcomes of their training. Physician burnout is being identified as a global crisis. It is the responsibility of all postgraduate training programs to identify strategies to prevent burnout in the postgraduate trainees and to empower them with skills to deal with it effectively throughout their work life [20]. This is an imperative need of the lower middle-income countries, where physician workload is highest, but physician wellbeing is long under-recognized [20]. Therefore, the objective of the present study was to determine the prevalence and associated factors of burnout, and it’s association with self-reported patient care practices, among postgraduate medical trainees in the district of Colombo, which has the highest number of teaching hospitals in Sri Lanka.

## Methods

A descriptive cross-sectional study was conducted among the total population of 278 postgraduate doctors registered with the Postgraduate Institute of Medicine (PGIM), Colombo. PGIM is the main institute for postgraduate medical education in Sri Lanka. It is a government-funded institute. All postgraduate doctors who had completed a minimum of 6 months of pre-MD (Doctor of Medicine) training in Clinical Medicine, Surgery, Gynaecology and Obstetrics, Paediatrics, Orthopaedics, Psychiatry, Anaesthesia and Emergency Medicine in training centres in Colombo district, during the period of 1.8.2016 to 31.8.2016 were included in the study. Postgraduates who were on maternity leave and on any other long leave for more than 1 month were excluded.

A self-administered questionnaire was used to obtain information on the burnout status, associated factors and self-reported patient care practices. It consisted of two parts. The first part included the Copenhagen Burnout Inventory (CBI) and questions on personal, family, work and training factors related to burnout. The second part assessed the frequency of engaging in six statements of self-reported patient care. These statements were used in a previous research study [18] to assess the association of burnout and self-reported patient care. The validity of these statements in the local setting was ensured using consensus of an expert panel.

CBI comprises of 3 scales. They are personal burnout scale, work-related burnout scale and client-related burnout scale. The personal burnout scale consists of six items [26], generated to evaluate the level of burnout in people, despite their occupational status [27]. Work-related burnout scale consists of seven items [26] that evaluate whether the person is attributing the fatigue and exhaustion experienced by him to his work [27]. Client-related burnout scale consists of six items [26] and it evaluates whether the person is attributing the fatigue and exhaustion experienced by him, to his work related with his clients such as patients, students, etc. [27] Validity and reliability of the CBI have been assessed by the developers of the tool, using the baseline and follow up data from the PUMA (Danish acronym for Project on Burnout, Motivation and Job Satisfaction) study. This was a prospective intervention study of human service sector workers. The internal reliability was very high with the Cronbach’s alpha for personal burnout, work-related burnout and client-related burnout being 0.87, 0.87, 0.85 respectively. The correlations between the three CBI scales were 0.72, 0.46 and 0.61 at the baseline. It has been validated and used in many other countries like Hongkong, Taiwan, New Zealand and Denmark [26, 28–30]. The CBI has been previously translated and validated in Sri Lanka [31] to measure burnout [27]. In the local validation study, the three subscales of the CBI have demonstrated high internal consistency, with Cronbach’s alpha values for personal burnout, work related burnout and client related burnout being 0.82, 0.82, and 0.81 respectively [31]. It has also shown high test-retest reliability (r = 0.89) [31].

Ethics approval for the present study was obtained from the Ethics Review Committee of Faculty of Medicine, University of Colombo. A pre-test of the questionnaire was done. Data collection was carried out by the principle investigator during the period from 1.8.2016 to 31.8.2016. Eligible participants were identified during lectures and seminars in each specialty training. They were approached individually. Informed written consent was obtained. The self-administered questionnaire was given to the postgraduate doctors who consented to participate. The questionnaires were collected after ensuring their completeness.

Data analysis was carried out using the SPSS (version 23) software. A mean score was calculated for each CBI scale from the scores of the individual items. A score of 50 was taken as cut off for the presence of burnout in each scale [21, 32]. Mean score, standard deviation and prevalence of burnout with the confidence intervals were calculated for each burnout type. Bivariate analysis was conducted using chi square test to determine the significance of the associations: burnout with different factors, and burnout with each statement of self-reported patient care. Statistically significant factors associated with burnout were further analysed using binary logistic regression.

## Results

The eligible number of postgraduate doctors was 278. The response rate was 88.1% (*n* = 245).

Age ranged from 28 to 40 years with a mean of 32.2 (SD 2.6) years. One hundred and fifty seven (64.1%) were males and 196 (80%) were married. Nearly half (48.2%, *n* = 118) had a monthly income in the range of Rs.90,000 – 120,000. Eighty (32.7%) were from clinical medicine, 41 (16.7%) from anaesthesia, 40 (16.3%) from surgery, 25 (10.2%) from paediatrics, and 20 (8.2%) from emergency medicine (Table 1).

**Table 1:** Socio demographic and work-related characteristics of the postgraduate doctors (*N* = 245)

Socio-demographic Characteristics	Number	Percentage
Age^a^ (*N* = 240)		
< 30 years	73	30.4
31–35 years	140	58.3
36–40 years	27	11.3
> 40 years	0	0.0
Sex		
Male	157	64.1
Female	88	35.9
Ethnicity		
Sinhala	192	78.4
Tamil	42	17.1
Muslim	11	4.5
Burgher	0	0.0
Other	0	0.0
Marital Status		
Married	196	80.0
Single	49	20.0
Monthly income		
< Rs. 90,000	28	11.4
Rs. 90,000 - 120,000	118	48.2
Rs. 121,000 – 150,000	89	36.3
Rs. 151,000 – 200,000	8	3.3
> Rs. 200,000	2	0.8
Specialty		
Medicine	80	32.8
Surgery	40	16.3
Paediatrics	25	10.2
Gynaecology & Obstetrics	18	7.3
Emergency Medicine	20	8.2
Orthopaedics	4	1.6
Psychiatry	17	6.9
Anaesthesia	41	16.7
Year of training		
1st year	67	27.4
2nd year	100	40.8
3rd year	78	31.8

^a^Missing values - 5

### Prevalence and the distribution of personal burnout, work-related burnout and client-related burnout among the postgraduate doctors

Table 2 provides descriptive statistics of scores obtained and the prevalence of each type of burnout.

**Table 2:** Prevalence and distribution of personal burnout, work related burnout and client related burnout among the postgraduate doctors

Type of Burnout	Mean Score (SD)	Prevalence^a^ (95% CI)
Personal burnout	48.6 (16.2)	41.6% (35.5 – 47.8%)
Work-related burnout	42.9 (15.9)	30.6% (24.8–36.4%)
Client -related burnout	31.8 (15.7)	8.9% (5.4–12.5%)

^a^A score of 50 was taken as the cut off for presence of burnout in each scale [21, 32]

The prevalence of personal burnout, work related burnout and client related burnout were 41.6% (95%CI = 35.5–47.8%), 30.6% (95%CI = 24.8–36.4%) and 8.9% (95%CI = 5.4–12.5%) respectively.

### Factors associated with personal burnout

Factors significantly associated with Personal burnout on bivariate analysis, were included as independent variables into a binary logistic regression model. This showed a R^2^ value of 45.8%. Table 3 provides a summary of the factors that were significantly associated with Personal burnout on logistic regression analysis.

**Table 3:** Factors that were significantly associated with personal burnout (N = 245)

Factor	Unadjusted			Adjusted		
	OR	95% CI	*P*	OR	95% CI	*P*
Personal Factors						
Female sex	2.3	1.3–3.9	0.002	3.6	1.8–7.3	< 0.001
Presence of chronic disease	2.7	1.3–5.8	0.006	4.0	1.5–11.0	0.007
High frequency of unhealthy habits	2.1	1.2–3.5	0.007	3.3	1.6–6.7	0.001
High frequency of healthy habits	0.2	0.1–0.4	< 0.001	0.2	0.1–0.5	< 0.001
Family related factors						
Parent/Parents being doctors	2.1	1.0–4.4	0.046	2.8	1.1–6.8	0.028
Occupation-related factors						
High home-work demands	2.7	1.4–5.0	0.002	2.9	1.3–6.2	0.008
High emotional demands	3.6	2.0–6.5	< 0.001	3.4	1.7–7.1	0.001
High Job resources	0.6	0.3–0.9	0.031			
Training-related factors						
Satisfied with relevance of training, in daily clinical practice	0.6	0.33–0.97	0.039			
Satisfied with accessibility to educational resources	0.6	0.34–0.97	0.036			
Satisfaction with opportunities for skill development	0.5	0.3–0.8	0.009	0.4	0.2–0.9	0.019
Satisfied with opportunities to develop clinical judgment	0.5	0.3–0.9	0.029			
Satisfied with time allocated for research	0.4	0.2–0.8	0.005			
Satisfied with overall training	0.4	0.2–0.8	0.006			
Good time management	0.4	0.2–0.7	0.001			
Frequent use of deep approaches when studying	0.3	0.2–0.5	< 0.001	0.2	0.1–0.5	< 0.001

Female postgraduate doctors were 3.6 times (OR = 3.6, 95%CI = 1.8–7.3) likely to be having personal burnout than males (*P* = < 0.001) while those having a chronic disease were 4.0 times (OR = 4.0, 95%CI = 1.5–11.0) likely to be having personal burnout (*P =* 0.007). Having high frequency of unhealthy habits (OR = 3.3, 95%CI = 1.6–6.7) and having parents who were doctors (OR = 2.8, 95%CI = 1.1–6.8) were also associated with personal burnout. Postgraduate doctors with high home-work demands (OR = 2.9, 95%CI = 1.3–6.2) and high emotional demands (OR = 3.4, 95%CI = 1.7–7.1) were more likely to be having personal burnout. The factors associated with a lower likelihood of personal burnout were, having high frequency of healthy habits (OR = 0.2, 95%CI = 0.1–0.5), being satisfied with opportunities for skill development from training programme (OR = 0.4, 95%CI = 0.2–0.9) and frequent use of deep approaches when studying (OR = 0.2, 95%CI = 0.1–0.5).

### Factors associated with work-related burnout

Binary logistic regression model to identify factors associated with work-related burnout had a R^2^ of 36.3%. Table 4 provides a summary of factors that were significantly associated with work-related burnout.

**Table 4:** Factors that were significantly associated with work-related burnout

Factor	Unadjusted			Adjusted		
	OR	95% CI	*P*	OR	95% CI	*P*
Personal factors						
Female sex	2.3	1.3–4.0	0.004	3.6	1.8–7.3	< 0.001
High frequency of unhealthy habits	2.0	1.1–3.7	0.019	2.8	1.4–5.8	0.005
High frequency of healthy habits	0.5	0.3–0.8	0.006			
Experience of a stressful life event during past 1 year	1.8	1.0–3.1	0.037			
Occupation-related factors						
High work load	2.2	1.3–3.8	0.005			
High home-work demands	4.3	2.2–8.1	< 0.001	5.5	2.6–11.8	< 0.001
High emotional demands	4.1	2.3–7.5	< 0.001	4.0	2.0–8.0	< 0.001
Training related factors						
Satisfied with accessibility to educational resources	0.57	0.33–0.99	0.046			
Satisfied with overall training	0.4	0.2–0.8	0.004			
Frequent use of deep approaches when studying	0.4	0.2–0.8	0.008	0.4	0.2–0.9	0.028

Females (OR = 3.6, 95%CI = 1.8–7.3), and those having a high frequency of unhealthy habits (OR = 2.8, 95%CI = 1.4–5.8) were more likely to be having work-related burnout. High home-work demands (OR = 5.5, 95%CI = 2.6–11.8) and high emotional demands (OR = 4.0, 95%CI = 2.0–8.0) were also positively associated with work-related burnout. Frequent use of deep approaches when studying (OR = 0.4, 95%CI = 0.2–0.9), was associated with a lower likelihood of work-related burnout.

### Factors associated with client-related burnout

Factors significantly associated with client-related burnout on bivariate analysis were included as independent variables into a binary logistic regression model. This showed a R^2^ value of 24.5%. Table 5 provides a summary of the factors that were significantly associated with client-related burnout.

**Table 5:** Factors that were significantly associated with client-related burnout

Factor	Unadjusted			Adjusted		
	OR	95% CI	*P*	OR	95% CI	*P*
Personal factors						
High frequency of healthy habits	0.3	0.1–0.8	0.014			
Occupation-related factors						
High emotional demands	5.6	2.2–14.1	< 0.001	4.0	1.5–10.6	0.005
High Job resources	0.4	0.1–1.0	0.044			
Training-related factors						
Satisfied with relevance of training, in daily clinical practice	0.41	0.17–0.99	0.044			
Being overall satisfied with specialty training	0.2	0.1–0.4	< 0.001	0.2	0.1–0.5	0.001
Good supervisor’s support	0.3	0.1–0.9	0.018			

Postgraduate doctors with high emotional demands were 4.0 times (OR = 4.0, 95%CI = 1.5–10.6) likely to be having client-related burnout. Being overall satisfied with specialty training (OR = 0.2, 95%CI = 0.1–0.5) was associated with a lower likelihood of having client related burnout.

### Association of suboptimal self- reported patient care practices with personal, work-related and client-related burnout

Frequency of engaging in six self-reported patient care practices was assessed. For each practice, a frequency of monthly or more was considered as ‘frequent’ practice, and a frequency less than once a month was considered as ‘infrequent’ practice. Association between frequent engagement in suboptimal patient care practices and the three burnout types were assessed. Table 6 provides a summary of the association of each self- reported patient care statement with personal, work-related and client-related burnout among the postgraduate doctors.

**Table 6:** Association of self-reported patient care practices with personal, work-related and client-related burnout among the postgraduate doctors (N=245)

Self-reported patient care statements	Personal burnout		Work-related burnout		Client-related burnout	
	Present	Absent	Present	Absent	Present	Absent
‘I discharged patients to make the ward more manageable’ (*N* = 204)^a^	43 (52.4%)	55 (45.1%)	29 (50.0%)	69 (47.3%)	12 (60.0%)	86 (46.7%)
	OR = 1.3 (0.8–2.3) *P* = 0.302		OR = 1.1 (0.6–2.0) *P* = 0.724		OR = 1.7 (0.7–4.4) *P* = 0.260	
‘I did not fully discuss treatment options or answer a patient’s/ patient’s parent’s questions’	34 (33.3%)	38 (26.6%)	25 (33.3%)	47 (27.6%)	11 (50.0%)	61 (27.4%)
	OR = 1.4 (0.8–2.4) *P* = 0.252		OR = 1.3 (0.7–2.3) *P* = 0.368		OR = **2.7 (1.1–6.4)** ***P*** **= 0.026**	
‘I ordered more laboratory or radiology tests because I was so busy’ (N = 204)^a^	16 (19.5%)	23 (18.9%)	12 (20.7%)	27 (18.5%)	8 (40.0%)	31 (16.8%)
	OR = 1.0 (0.5–2.1) *P* = 0.906		OR = 1.1 (0.5–2.5) *P* = 0.719		OR = **3.3 (1.2–8.7)** ***P*** **= 0.028**	
‘I did not treat a patient’s pain in a timely manner’	21 (20.6%)	20 (14.0%)	15 (20.0%)	26 (15.3%)	10 (45.5%)	31 (13.9%)
	OR = 1.6 (0.8–3.1) *P* = 0.172		OR = 1.4 (0.7–2.8) *P* = 0.363		OR = **5.2 (2.0–13.0) P = < 0.001**	
‘I did not communicate important information during handoff to my colleague’	22 (21.6%)	23 (16.1%)	16 (21.3%)	29 (17.1%)	9 (40.9%)	36 (16.1%)
	OR = 1.4 (0.7–2.7) *P* = 0.274		OR = 1.3 (0.7–2.6) *P* = 0.426		**OR = 3.6 (1.4–9.0)** ***P*** **= 0.01**	
‘I did not discuss a patient’s treatment plan with the patient’s appropriate nursing or ancillary staff’	38 (37.3%)	40 (28.0%)	27 (36.0%)	51 (30.0%)	11 (50.0%)	67 (30.0%)
	OR = 1.5 (0.9–2.6) *P* = 0.124		OR = 1.3 (0.7–2.3) *P* = 0.353		OR = 2.3 (1.0–5.6) *P* = 0.055	

^a^Anaesthesia trainees and Emergency Medicine trainees undergoing Anaesthesia appointment were excluded since they are not involved in these practices

ORs and *p* values shown in bold print indicate patient care practices that were significantly (*p* < 0.05) associated with burnout

Frequent practice of the self-reported patient care statements ‘I did not fully discuss treatment options or answer a patient’s/ patient’s parent’s questions’ (OR = 2.7;95%CI = 1.1–6.4), ‘I ordered more laboratory or radiology tests because I was so busy’ (OR = 3.3;95%CI = 1.2–8.7), ‘I did not treat a patient’s pain in a timely manner’ (OR = 5.2;95%CI = 2.0–13.0) and ‘I did not communicate important information during handoff to my colleague’ (OR = 3.6;95%CI = 1.4–9.0), were significantly associated with client-related burnout.

## Discussion

This study was conducted in the Colombo district, which has the largest number of postgraduate doctors training in various specialties of medicine, in Sri Lanka. Unlike many previous studies on burnout on postgraduate doctors, present study included eight main medical specialties (Clinical Medicine, Surgery, Gynaecology and Obstetrics, Paediatrics, Orthopaedics, Psychiatry, Anaesthesia and Emergency Medicine). Selection bias was minimised by including the total population. Burnout was measured using the Copenhagen Burnout Inventory, which was a locally validated tool to measure burnout [27, 31]. The CBI, factors associated with burnout and self-reported patient care statements for postgraduate doctors were used after ensuring consensual validity of each component through the assessment of a panel of experts.

The present research study reported a relatively high personal burnout and work-related burnout within the range of 27–75%, which was reported in previous studies [3–5, 10, 21, 33–36]. Yet the client-related burnout was relatively low, the most likely reason being the postgraduates being adapted to high patient loads from the onset of their career and therefore not perceiving it as a burden. The high prevalence highlights that burnout is a significant problem in this population. It can prevent them from achieving the maximum out of their training and therefore, early preventive measures should be implemented.

In the current study, the personal factors: being a female, having a chronic disease and high frequency of unhealthy habits had a positive association with burnout. High frequency of healthy habits was negatively associated with burnout. Previous evidence on gender and burnout is inconclusive [7, 10, 17, 21]. These contrasting associations with gender seen among various populations of postgraduate doctors can be due to the variations in gender roles and cultural differences. Association of burnout with having chronic diseases is previously shown in other occupational groups [37]. The presence of a chronic disease and related mental and physical burden can be a cause as well as a consequence of burnout. The exposure to chronic work stress can leads to development of non-communicable diseases. Similar to the present study, unhealthy habits like smoking and drug use [8], difficulty in eating healthy [13] and consumption of alcohol [9] were previously shown to be positively associated with burnout. It was negatively associated with healthy practices like meditation, relaxation, massage or other alternatives [8] and fulfilling the physical activity guidelines [38], similar to the present study. These findings reiterate the importance of incorporating practical guidance on healthy lifestyle to the teaching curricula of postgraduate education. This will equip the future professionals with competencies to promote and maintain their physical health. It will also reduce work related stress and burnout. This is of paramount importance when considering the low priority given currently to the wellbeing of the doctors in the lower middle-income countries [20].

Of the family-related factors, having parents who were doctors had a positive association with burnout in the current study. Previous research has failed to identify an association between employment of parents and burnout [39]. This observation in the present study can be due to the increased pressure the postgraduate doctors might be receiving from their parents. It can also be due to society’s expectations to continue their postgraduate education. The trainees themselves may also have had high expectations since childhood by taking their parents as their role models, which might be leading to burnout.

Of the work-related factors, home-work demands and emotional demands were positively associated burnout. Similar results were observed in previous research studies among postgraduate doctors, on association of home-work demands and burnout [6, 8, 12, 13, 40]. Postgraduate doctors undergo training, knowledge gathering and regular ward work. When their home demands also increase, they may get over-burdened, which will make them more susceptible to burnout. On the other hand, the doctors who are having burnout, due to their exhaustion and negative thoughts may be rating their home-work demands as unduly high. Results similar to the present study were observed in previous research among postgraduate doctors, on emotional demands and burnout [14, 41–43]. Working under emotionally taxing situations is known to lead to stress and burnout. Association shown between burnout and work-home demands and emotional demands can be explained by the Job Demands-Resources Model of Burnout [44], which explains the basis of increase in work demands leading to increase in exhaustion. Identification of such work demands specific to postgraduate doctors would facilitate the planning of interventions, to prevent and manage burnout in this population.

Of the training-related factors: being satisfied with opportunities available for skill development in the training programme, frequency of utilizing deep approaches when studying and being satisfied with the overall specialty training were associated with less burnout. Opportunities for skill development in the specialty training will equip the trainee with the competencies and confidence for successful completion of his clinical responsibilities. This will make them less susceptible for burnout. Frequent utilization of deep approaches during studying will help to retain more information, which can be used during daily practice and examinations. Therefore, the postgraduate doctor will not be overburdened with last minute studying and would be less susceptible to burnout. Less burnout was observed in those who were satisfied with the overall specialty training in previous research studies among postgraduate doctors [10, 35]. It is likely that those who are satisfied with the training have gained the necessary skills and knowledge needed to engage in the training programme without getting burnout. In contrast, those who were unsatisfied that their training needs were not met, were more likely to have burnout. These training related factors need to be taken into consideration by the postgraduate programme planners. It is important that a module on personal development is included into the training programmes. This should focus on competencies like study skills and handling stress.

In the present research, the frequent practice of the self-reported patient care statements: ‘I did not fully discuss treatment options or answer a patient’s/ patient’s parent’s questions’, ‘I ordered more laboratory or radiology tests because I was so busy’, ‘I did not treat a patient’s pain in a timely manner’ and ‘I did not communicate important information during handoff to my colleague’, were positively associated with burnout. Similar results were observed in previous studies on postgraduate doctors [3, 18]. These findings show that when the postgraduate doctors are experiencing burnout, it affects the patient care provided by them, leading to suboptimal patient care practices. This finding highlights that burnout in postgraduate doctors results in negative consequences towards their patients.

### Limitations of the study

Being a cross-sectional study, it was not possible to check for temporality of the associations. Therefore, the factors identified to be associated with burnout may be causes, effects or an outcome arising out of the same root cause as that of burnout. During analysis, arbitrary cut offs were used to dichotomize certain variables on associated factors. Cut-offs for the CBI were not available for Sri Lankan postgraduate doctors. Therefore, according to previous research, a score of 50 or more was considered as the cut off for all three burnout scales [21, 32]. The factors associated with burnout that were identified were measured as perceived by the postgraduates. This might have led to an increase in the magnitude of the associations that were observed. The study was limited to the district of Colombo. However, the findings can be reasonably representative of the postgraduate doctors training throughout Sri Lanka, since other teaching hospitals throughout the country have similar circumstances, patient loads and facilities.

## Conclusions

Most postgraduate doctors in Colombo have high personal and work-related burnout. Yet the client-related burnout is less. The factors associated with burnout need to be addressed by the programme managers of the postgraduate courses. Preventive measures should be introduced to reduce burnout among future postgraduate trainees.

### Recommendations

In all postgraduate training programmes, mental health promotion of the trainees should be made a high priority. A training module on personal and professional development should be introduced at the beginning and reinforced during the postgraduate training. This should include skills on time management, management of difficult patients, managing home-work demands, communication, stress management, etc. Mentoring and counselling systems should be developed and strengthened.

## Data Availability

Data pertaining to this study is available from the corresponding author on reasonable request.

## Abbreviations

CBI: Copenhegan Burnout Inventory
CI: Confidence Interval
MD: Doctor of Medicine
OR: Odds Ratio
PGIM: Postgraduate Institute of Medicine, University of Colombo
PUMA: Danish acronym for Project on Burnout, Motivation and Job Satisfaction
R^2^: R squared (Coefficient of determination)
SD: Standard deviation
SPSS: Statistical Package for the Social Sciences
